# High Glucose Alters Retinal Astrocytes Phenotype through Increased Production of Inflammatory Cytokines and Oxidative Stress

**DOI:** 10.1371/journal.pone.0103148

**Published:** 2014-07-28

**Authors:** Eui Seok Shin, Qiong Huang, Zafer Gurel, Christine M. Sorenson, Nader Sheibani

**Affiliations:** 1 Department of Ophthalmology and Visual Sciences, University of Wisconsin School of Medicine and Public Health, Madison, Wisconsin, United States of America; 2 Department of Pediatrics, University of Wisconsin School of Medicine and Public Health, Madison, Wisconsin, United States of America; 3 McPherson Eye Research Institute, University of Wisconsin School of Medicine and Public Health, Madison, Wisconsin, United States of America; Case Western Reserve University, United States of America

## Abstract

Astrocytes are macroglial cells that have a crucial role in development of the retinal vasculature and maintenance of the blood-retina-barrier (BRB). Diabetes affects the physiology and function of retinal vascular cells including astrocytes (AC) leading to breakdown of BRB. However, the detailed cellular mechanisms leading to retinal AC dysfunction under high glucose conditions remain unclear. Here we show that high glucose conditions did not induce the apoptosis of retinal AC, but instead increased their rate of DNA synthesis and adhesion to extracellular matrix proteins. These alterations were associated with changes in intracellular signaling pathways involved in cell survival, migration and proliferation. High glucose conditions also affected the expression of inflammatory cytokines in retinal AC, activated NF-κB, and prevented their network formation on Matrigel. In addition, we showed that the attenuation of retinal AC migration under high glucose conditions, and capillary morphogenesis of retinal endothelial cells on Matrigel, was mediated through increased oxidative stress. Antioxidant proteins including heme oxygenase-1 and peroxiredoxin-2 levels were also increased in retinal AC under high glucose conditions through nuclear localization of transcription factor nuclear factor-erythroid 2-related factor-2. Together our results demonstrated that high glucose conditions alter the function of retinal AC by increased production of inflammatory cytokines and oxidative stress with significant impact on their proliferation, adhesion, and migration.

## Introduction

Astrocytes (AC) are macroglial cells with important role in retinal vascular development, and provide physical support and nutrient for neurons in the central nerveous system. AC also have foot processes that envelop retinal endothelial cells (EC) in blood vessels to maintain blood retina barrier (BRB) [1], [2]. In addition, AC regulate fluid and electrolyte balance by expressing channel proteins including water channel proteins, aquaporin 4 and the potassium channel Kir4.1 at the luminal spaces of their end foot processes [3], [4]. Astrocytes regulate blood barrier function by secreting growth factors such as transforming growth factor-β (TGF-β), glial-derived neurotrophic factor (GDNF), basic fibroblast growth factor (bFGF) and angiopoetin 1(ANG1) [5], [6]. Furthermore, AC secretion of sonic hedgehog (Shh) enhances barrier function and decrease inflammatory mediators of the endothelium [7].

The structural integrity of BRB is crucial in the pathogenesis of retinal vascular disease including diabetic retinopathy (DR). Pathological conditions affect physiology of cellular components of BRB including EC, pericytes (PC) and AC, leading to the breakdown of BRB structures. During diabetes, vascular cells are affected by the high glucose environment. High glucose conditions promote migration of retinal EC through activation of signaling pathway mediated by Src, PI3K/Akt1/eNOS and ERK [8]. In contrast, high glucose conditions increased apoptosis of retinal PC by activation of protein kinase C-δ (PKC-δ) and Src homology-2 domain-containing phosphatase-1(SHP-1) [9]. Diabetic conditions have been shown to affect retinal AC. In diabetic retina, morphology and viability of retinal AC are compromised compared with normal conditions [10], [11]. Although changes in AC contribute to alterations in retinal vascular structures and function under hyperglycemic conditions, the cellular mechanism leading to dysfunction of retinal AC remain poorly defined.

High glucose conditions impair various cellular functions including adhesion, migration, proliferation and apoptosis. Production of inflammatory mediators and oxidative stress are identified as key elements in high glucose mediated vascular cell dysfunction [9], [12], [13], [14]. We recently showed activation of STAT1, through production of inflammatory cytokines in response to high glucose, is responsible for increased expression of proapoptotic protein Bim, increased oxidative stress, and retinal pericyte apoptosis [15].To respond to oxidative stress, protective mechanisms are implicated in cells exposed to various stress including high glucose conditions. The transcription of genes to protect from oxidative stress is regulated by a redox-sensitive transcription factor, nuclear factor-erythroid 2-related factor-2 (Nrf2) [16]. Activation of Nrf2 by oxidative stress results in its translocation into the nucleus and activation of transcription of antioxidant genes including heme oxygenase-1 (HO-1) and peroxiredoxin-2 (Prdx2) [16]. Nrf2 pathway is activated to protect AC from cell death mediated by oxidative stress in neurodegenerative diseases [17]. However, the effect of high glucose conditions on the activation of this defense system in retinal AC has not been previously determined.

We have previously described a novel method for isolation and culture of retinal AC from wild type and transgenic mice [18]. Although high glucose conditions did not result in apoptosis of AC, they did have a significant impact on production of reactive oxygen species (ROS), production of inflammatory mediators, activation of NF-κB, and attenuation of AC migration and capillary morphogenesis of retinal EC. These defects in AC were reversed in the presence of anti-oxidant N-acetylcysteine (NAC). We also determined that high glucose conditions activated the protective intracellular mechanisms via enhanced translocation of Nrf2 into the cell nucleus, and increased expression of anti-oxidant proteins. Thus, high glucose conditions have significant impact on AC proliferation, adhesion, and migration through increased production of inflammatory cytokines and oxidative stress.

## Materials and Methods

### Cell Culture Conditions

Mouse retinal astrocytes (AC) were isolated and cultured as previously described [18]. These cells were isolated from Immorto mice backcrossed to C57BL/6J for at least 10 generation. These cells express a temperature sensitive SV-40 large T-antigen, which allows their proliferation at permissive 33°C temperature. All animal experiments were conducted in accordance with the Association for Research in Vision and Ophthalmology statement for the use of Animals in Ophthalmic and Vision Research and were approved by the Institutional Animal Care and use Committee of the University of Wisconsin School of Medicine and Public Health. The cells were plated on 1% gelatin-coated 60 mm dishes and cultured in Dulbecco's Modified Eagle's Medium (DMEM) containing 10% fetal bovine serum (FBS), 2 mM L-glutamine, 2 mM sodium pyruvate, 20 mM HEPES, 1% nonessential amino acids, 100 µg/ml streptomycin, 100 U/ml penicillin, freshly added heparin at 55 U/ml (Sigma, St. Louis, MO), endothelial growth supplement 100 µg/ml (Sigma), and murine recombinant interferon-γ (R & D, Minneapolis, MN) at 44 units/ml. Cells were maintained at 33°C with 5% CO_2_. The growth medium contained either 5.7 mM D-glucose (normal glucose, NG), 40.7 mM D-glucose (hyperglycemia/high glucose, HG), or 5.7 mM D-glucose+35 mM L-glucose as high osmolarity control (NG+L-glu). These glucose conditions are in line with levels of glucose detected in blood of diabetic mice, and have been used to study the impact of HG on retinal endothelial cells and pericyte function [8], [15]. Cells were maintained in growth medium for 5 days prior to each experiment, and the medium was changed every other day. Three different isolation of AC were used in the studies presented here and each experiment was repeated at least once.

### Cell Proliferation and Apoptosis Assays

The rate of DNA synthesis was determined using EdU DNA labeling (Click-iT EdU Flow Cytometry kit, Invitrogen, Carlsbad, CA). Cells cultured under different glucose conditions were incubated with 10 µM EdU for 1 h. Following incubation, cells were removed using cell dissociation solution (Sigma) and analyzed by FACScan caliber flow cytometer as recommended by the supplier (Becton-Dickinson, Franklin Lakes, NJ). Positive cells were calculated as a percentage of total cell number. The rate of apoptotic cell death was assessed by TdT-dUTP Terminal Nick-End Labeling (TUNEL) staining. Cells were cultured under different glucose conditions in fibronectin coated chamber slides (2 µg/ml in phosphate buffered saline; PBS) for five days. Cells were then washed twice with PBS, fixed with 4% paraformaldehyde for 20 min and washed with PBS twice. Cells were permeabilized with 0.5% Triton X-100 in PBS for 10 min. Apoptotic cell death was assessed using Click-iT TUNEL Alexa Fluor Imaging Assay (Invitrogen, Carlsbad, CA,) as recommended by the manufacturer. Positive cells were counted under fluorescence microscope and the percentage of apoptotic cells relative to the total number of cells was calculated.

### Transwell Migration Assays

Cell migration was assessed in transwell assays as previously described [8]. Briefly, transwell inserts (8-µm pore size, 6.5-mm membrane; Costar, Lowell MA) were coated with fibronectin (2 µg/ml) in PBS on the bottom side at 4°C overnight. The next day, inserts were rinsed with PBS, blocked in PBS containing 2% BSA for 1 h at room temperature, and washed with PBS. Cells that have been cultured in medium with different glucose conditions for 5 days were removed by trypsin-EDTA, counted, and resuspended at 1×10^6^ cells/ml in serum-free medium. Inserts were placed in 24-well plates (Costar) containing 0.5 ml serum-free medium, and 0.1 ml cell suspension was then added to the top of the inserts. Cells were allowed to migrate through the membrane for 4 h at 37°C. To examine the effect of PDGF-AA or PDGF-BB on migration, transwell inserts were placed in 24-well dishes containing 0.5 ml serum-free medium or serum-free medium containing PDGF-AA or PDGF-BB (50 ng/ml; PeproTech, Rocky Hill, NJ). Following incubation, the cells on top of the filter were scraped off using a cotton swab. The membrane was fixed in 4% paraformaldehyde and stained with hematoxylin and eosin. The inserts were removed, mounted on a slide, cell side up, and the number of cells migrated to the bottom of the filter was determined by counting 10 high-power fields (×400). To examine the migration of EC, 1 × 10^5^ cells in 100 µl of medium were added to the top of each Transwell membrane. Transwell inserts were placed in 24-well dishes containing conditioned medium collected from RAC cultured under different glucose conditions for five days. Cells were allowed to migrate through the membrane for 3 h at 37°C. Following incubation, number of migrated cells was determined as described above.

### Indirect Immunofluorescence Staining

Retinal AC (1×10^4^) were plated on glass cover slips coated with 2 µg/ml of fibronectin. Cells were incubated under different glucose conditions for five days. Cells were rinsed with PBS, fixed with 3% paraformaldehyde (PFA) for 10 min on ice, washed twice with PBS. Cells were incubated in PBS containing 0.25% Triton X-100 for 15 minutes at room temperature for permeabilization. After washing cells with TBS twice, cells were incubated with anti-vinculin (1∶100; Sigma), FITC-phalloidin (1∶200; Sigma), and DAPI (Invitrogen, D1306; 10 µg/ml) for 40 min at 37°C. For analyzing cellular location of Nrf2, Anti-Nrf2 (H-300; sc-13032, Santa Cruz Biotechnology, Santa Cruz, CA) was also used. After washing three times with PBS, cells were incubated with appropriate CY3-conjugated secondary antibodies (Jackson ImmunoResearch, West Grove, PA) at 37°C for 40 min. Cells were washed three times with PBS, mounted, and photographed using a Zeiss fluorescence microscope (Axiophot, Zeiss, Germany) equipped with a digital camera. Coherency of stress fiber, number of focal adhesions and intensity of nuclear signals were quantified using Image J software (NIH; http://rsb.info.nih.gov/ij).

### Cell Adhesion Assays

The retinal AC adhesion to various extracellular matrix proteins was determined as recently described. Briefly, 96 well plates (Maxisorb, Nunc) were coated with various concentrations of fibronectin (FN), vitronectin (VN), collagen-I(Col-I) and laminin (LM) (BD Biosciences, San Jose, CA) prepared in TBS (Tris-buffered saline, 20 mM Tris-HCl pH 7.6, 150 mM NaCl) with 2 mM Ca^2+^ and 2 mM Mg^2+^ (Ca/Mg) overnight at 4°C. The next day the plates were rinsed four times with TBS containing Ca/Mg, blocked for 1 h with 200 µl of 1% BSA prepared in TBS with Ca/Mg for at least 1 h at room temperature. Cells cultured under different glucose conditions for 5 days were removed using cell dissociation solution (Sigma, St. Louis, MO), washed once with TBS, and resuspended in HEPES buffered saline (25 mM HEPES pH 7.6; 150 mM NaCl) containing 4 mg/ml of BSA at 5×10^5^ cells/ml. Blocking solution was then removed from the coated plates and each well received 50 µl of TBS with Ca/Mg and 50 µl of cell suspension in triplicates. Cells were then allowed to adhere for 90 min at 37°C and non-adherent cells were removed by gently washing the wells with 200 µl of TBS with Ca/Mg until no cells were left in control wells coated with BSA. The number of adherent cells in each well was quantified by measuring the cellular phosphatase activity as previously described [19]. All samples were done in triplicate.

### Formation of Retinal AC Network and Capillary Morphogenesis of Retinal EC on Matrigel

We previously showed that retinal AC organize into a network when plated on Matrigel similar to retinal EC [18]. We next determined the ability of cells cultured under different glucose conditions to organize into three dimensional networks. Approximately, 2×10^5^ cells in 2 ml of growth medium were plated in a 35 mm dish coated with 0.5 ml of 10 mg/ml Matrigel (BD Biosciences, Bedford, MA), incubated at 37°C for 16 to 24 h, and photographed in a digital format. For quantitative assessment, the mean number of branch points in five high power fields (×100) was determined.

Mouse retinal EC were prepared and cultured as previously described by us [20]. For analyzing capillary morphogenesis of retinal EC, cells were removed by trypsin-EDTA, washed with DMEM containing 10% FBS, and resuspended at 2×10^5^ cells/ml in conditioned medium collected from retinal AC incubated under different glucose conditions. Cells (2 × 10^5^) in 2 ml were applied to the Matrigel-coated plates, incubated at 37°C, photographed after 18 h using a Nikon microscope in digital format.

### FACScan Analysis

Cells cultured under different glucose conditions were washed once with PBS containing 0.04% EDTA and collected using cell dissociation solution (Sigma). Cells were washed once with DMEM containing 10% FBS and blocked in TBS with 1% goat serum for 20 min on ice. Cells were incubated with specific primary antibodies for 30 min on ice. The anti-cleaved caspase 3 antibody (Cell signaling, Danvers, MA), anti-GFAP (Dako, Carpentaria, CA), anti-α5β1 integrin (MAB1976Z), or anti-αvβ3 integrin (MAB1999) (Millipore, Temecula, CA) was prepared in TBS with 1% BSA at 2 µg/ml. Following incubation, cells were then washed twice with TBS with 1% BSA, and incubated with appropriate FITC-conjugated secondary antibody for 30 min on ice. The stained cells were washed twice with TBS with 1% BSA and resuspended in 0.5 ml of TBS with 1% BSA, and analyzed by FACScan caliber flow cytometer (Becton-Dickinson, Franklin Lakes, NJ). Cells incubated with secondary antibodies in the absence of primary antibodies were used as negative control. The average mean fluorescence intensities were used for quantitative comparisons.

### Western Blot Analysis

Approximately, 2×10^5^ cells were plated in 60-mm tissue culture plates and cultured under different glucose conditions. Cells were washed twice with cold PBS, lysed in 0.1 ml of lysis buffer (20 mM Tris Ph 7.4, 2 mM EDTA, 25 mM NaF, 1 mM Na_3_VO_4_, 1% Triton X-100, 1% NP-40, 0.1% SDS and a protease inhibitor cocktail (Roche Biochemicals, Indianapolis, IN)) and briefly sonicated. The lysates were centrifuged and protein concentrations were determined using the BCA protein assay kit (Pierce, Rockford, IL). For conditioned medium, cells were rinsed once with serum free medium and incubated with serum free medium (complete medium with different conditions of glucose but no serum) for two days. The conditioned medium was collected, and clarified by centrifugation. The appropriate volume of protein samples, corrected for cell number, were mixed with sufficient volume of 6X SDS sample buffer and analyzed by 4-20% SDS-PAGE (Invitrogen). Proteins were transferred to nitrocellulose membrane, blocked in TBS containing 5% BSA and 5% non-fat milk, and incubated with appropriate primary antibodies at 4°C overnight. After washing with TBS containing 0.1% Tween 20, the blots were incubated with appropriate HRP-conjugated secondary antibodies (1∶10,000; Jackson Immunoresearch Laboratories) and developed using ECL (Amersham). The same blot was re-probed with a monoclonal antibody to β-actin (Sigma) to verify equal protein loading in lanes with cell lysates. The following primary antibodies were used: p-Src (Tyr418), p-p38 (Thr180/Tyr182), p38, p-Akt1 (ser 473), Akt1, p-ERK, ERK and HO-1 (Cell Signaling, Danvers, MA), p-JNK1, and JNK1(R&D System, Minneapolis, MN), Prdx2 (Pierce Biotechnology, Rockford, IL), p-p65 (sc-33039) and p65 (c-20; sc-372) (Santa Cruz Biotechnology), thrombospondin-1 (TSP1) (A6.1, Neo Markers, Fremont, CA), iNOS and TSP2 (BD Biosciences). For quantitative comparisons average band intensities relative to total proteins and/or β-actin were used using Image J software.

### Immunoprecipitation of Fyn

To determine the levels of active Src family member Fyn in retinal AC under different glucose conditions, equal amount of cell lysates were used for immunoprecipitation assay. The lysates were pre-cleared with protein G-agarose (Sigma) and incubated with mouse anti-Fyn antibody (sc-16; Santa Cruz Biotechnology) overnight at 4°C. Following incubation with the primary antibody, 50 µl of anti-mouse protein G agarose was added to the sample and incubated for 2 h at 4°C.The beads were centrifuged at 2,000 rpm for 5 min, and washed three times with lysis buffer. The beads were resuspended in 6x SDS sample buffer and boiled for 10 min. The immunoprecipitation eluates were separated on SDS-PAGE and transferred to a nitrocellulose membrane. The blot was probed for the activated, phosphorylated form of Fyn by using a specific antibody to the p-Src (Tyr418) (Cell signaling), and for total Fyn using anti-Fyn antibody (sc-16; Santa Cruz Biotechnology).

### RNA Purification and Real Time qPCR Analysis

Total RNA from retinal AC was extracted using mirVana PARIS kit (Invitrogen). The cDNA synthesis was performed from 1 µg of total RNA using Sprint RT Complete-Double PrePrimed kit (Clontech, Mountain View, CA). One µl of each cDNA (dilution 1∶10) was used as template in qPCR assays, performed in triplicate of three biological replicates on Mastercycler Realplex (Eppendorf) using the SYBR-Green qPCR Premix (Clontech). Amplification parameters were as follows: 95°C for 2 min; 40 cycles of amplification (95°C for 15 sec, 60°C for 40 sec); dissociation curve step (95°C for 15 sec, 60°C for 15 sec, 95°C for 15 sec). Primer sequences for TNF-α were 5′-ACCGTCAGCCGATTTGCTAT-3′ (forward) and 5′-TTGACGGCAGAGAGGAGGTT-3 (reverse). For IL-1β, 5′- GTTCCCATTAGACAA CTGCACTACA-3′ (forward), and 5′- CCGACAGCACGAGGCTTTT-3′ (reverse); For MCP-1, 5′-GTCTGTGCTGACCCC AAGAAG-3′ (forward), and 5′-TGGTTCCGATCCAGG TTTTTA-3′ (reverse). Standard curves were generated from known quantities for each of the target gene of linearized plasmid DNA. Ten times dilution series were used for each known target, which were amplified using SYBR-Green qPCR. The linear regression line for ng of DNA was determined from relative fluorescent units (RFU) at a threshold fluorescence value (Ct) to quantify gene targets from cell extracts by comparing the RFU at the Ct to the standard curve, normalized by the simultaneous amplification of RpL13a, a housekeeping gene. Primer sequences for RpL13a were 5′-TCTCAAGGTTGTTC GGCTGAA-3′ (forward) and 5′-CCAGACGCCCCAGGTA-3′ (reverse).

### Determination of Oxidative Stress

Cells (0.1 ml of 1×10^5^ cells/ml) were plated on fibronectin (2 µg/ml)-coated 4-well chamber slides. After attachment, cells were cultured in medium under different glucose conditions for 5 days. The level of cellular reactive oxygen species (ROS) was assessed using dihydroethidium staining (DHE; Invitrogen), as previously described [21]. For quantitative assessments, the images were analyzed using Image J software. Values were obtained from each cells captured in 5 high power fields (x400). More than sixty cells per each condition were analyzed.

### Statistical Analysis

Statistical differences between control and treated samples were evaluated with student's unpaired *t-*test (2-tailed) or one-way ANOVA with post hoc Bonferroni test for multiple comparisons. Mean ±SE are shown. *p* values ≤ 0.05 were significant.

## Results

### The Effects of High Glucose on Retinal AC Proliferation and Apoptosis

**Figure 1:**
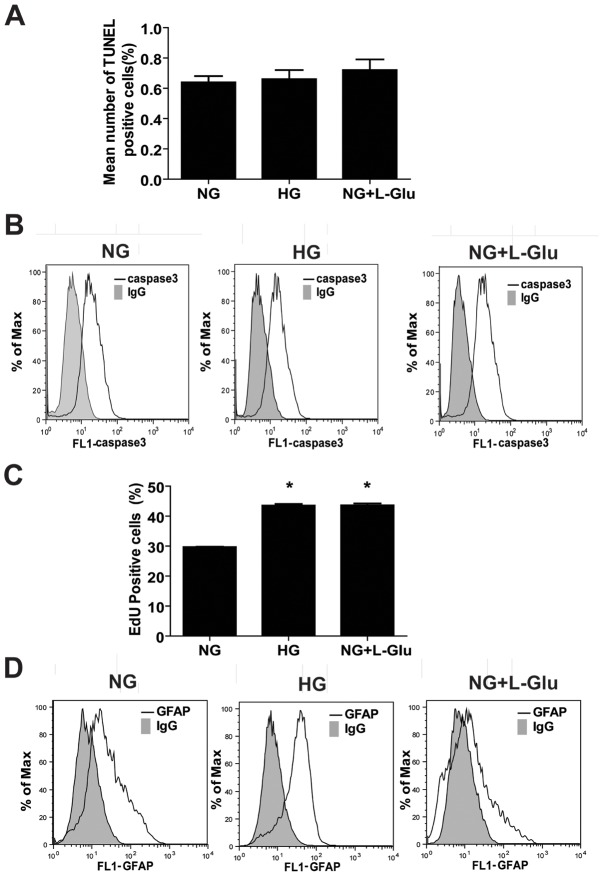
High glucose conditions had no effect on apoptosis of retinal AC but enhanced their DNA synthesis and GFAP expression. (A) The rate of apoptosis was determined by TdT-dUTP Terminal Nick-End Labeling (TUNEL) staining. Positive cells were counted using a fluorescence microscope and calculated as percentage of total cell number per field. (B) Level of cleaved caspase-3 was determined by FACS analysis. Shaded areas show staining in the absence of primary antibody. (C) The percentage of retinal AC undergoing active DNA synthesis was determined using FACScan flow cytometery. Data are presented as Mean ± SEM. *p<0.05; *n* = 3 (NG vs. HG and NG vs. NG+L-Glu). EdU is 5-ethynyl-2′-deoxyuridine. (D) Level of GFAP in retinal AC was determined by FACS analysis. High glucose conditions increased the mean fluorescence intensity of GFAP by 1.5-fold in retinal AC compared with normal glucose and osmolarity control conditions (P<0.05, n = 3). Shaded areas show staining in the absence of primary antibody.

We determined the rate of apoptosis in retinal AC cultured under different glucose conditions as detailed in methods. Apoptotic cell death was determined by TdT-dUTP Terminal Nick-End Labeling (TUNEL) assay. Figure 1A shows that the retinal AC under high glucose or osmolarity control conditions exhibited a similar rate of apoptosis as those cells cultured under normal glucose conditions. Cleaved caspase-3 level in retinal AC under different glucose conditions was also determined by FACS analysis. Different glucose conditions also did not affect the level of cleaved caspase-3 in retinal AC (Fig. 1B). The average mean fluorescence intensities were not significantly different (NG: 18.2±3.2 vs. HG: 14.7±1.5 vs. NG+L-Glu: 16.9±2.0; P>0.05; n = 3). Thus, high glucose conditions had minimal effect on apoptosis of retinal AC.

We next asked whether high glucose affects the rate of DNA synthesis in retinal AC cultured under various glucose conditions. Figure 1C shows the mean percentage of positive cells labeled with EdU under different glucose conditions. We observed a significant increase in the percentage of cells undergoing active DNA synthesis under high glucose or osmolarity control conditions compared to the cells cultured under normal glucose conditions. Thus, osmotic stress was associated with increased percentage of retinal AC undergoing active DNA synthesis.

Glial fibrillary acidic protein (GFAP) is a marker of AC and known to be up-regulated during diabetes and neuronal damage [22]. We next determined the level of GFAP in retinal AC under various glucose conditions. High glucose conditions increased GFAP level by approximately 1.5-fold in retinal AC compared with normal glucose and osmolarity control (Fig. 1D). The average mean fluorescence intensities were NG: 20.8±1.5 vs. HG: 32.5±2.6 (P<0.05) or NG+L-Glu: 18.7±2.4 (P>0.05); n = 3. These results are consistent with increased expression of GFAP detected in retinal sections of diabetic mice [23], [24].

### Attenuation of Retinal AC Network Formation under High Glucose Conditions

**Figure 2:**
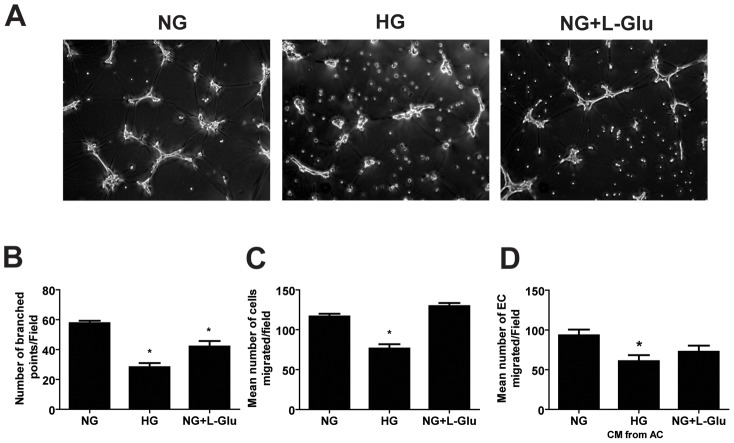
Attenuation of retinal AC migration and network formation in Matrigel under high glucose conditions. (A) High glucose conditions inhibited the organization and network formation of retinal AC in Matrigel. (B) The quantitative assessment of the data. Data are the mean number of branch points from 5 high-power fields (×100) ± SEM. *p<0.05; n = 5 (NG vs. HG and NG vs. NG+L-Glu). (C) Transwell migration of retinal AC under different glucose conditions. High glucose conditions significantly inhibited migration of retinal AC compared with normal glucose and osmolarity control. Data are presented as mean ± SEM. n = 3,*p<0.05 (NG vs. HG). (D) Conditioned medium was collected from retinal AC incubated under normal glucose, high glucose, or osmolarity control for five days. The effects of these conditioned medium on the migration of retinal EC was determined in a transwell migration assay. Please note a significant decrease in migration of retinal EC incubated with conditioned medium from retinal AC cultured under high glucose conditions. Data are presented as mean ± SEM. n = 3; *p<0.05 (NG vs. HG)

Prior to retinal vascularization, retinal AC migrate from the optic nerve to create a scaffold-like network which is utilized by EC to establish the primary retinal vascular network. We have previously shown that, like retinal EC, retinal AC organize into a three dimensional-like network when plated on Matrigel [18]. We next asked whether high glucose conditions affect retinal AC to organization into a network on Matrigel. Figure 2A shows that retinal AC under normal glucose conditions readily organized on Matrigel forming a network. In contrast, this ability was attenuated under high glucose or osmolarity control conditions. The quantitative assessment of the data is shown in Figure 2B. Thus, exposure of retinal AC to osmotic stress has a significant impact on their ability to organize into a network, which may impact the integrity of the retinal vascular network.

### High Glucose Attenuates Retinal AC Migration

The migratory properties of retinal AC were assessed using a transwell assay. Figure 2C demonstrates the migratory activity of AC cultured under different glucose conditions. We observed a significant decrease in the number of cells migrated through the transwell under high glucose conditions, compared with normal or osmolarity control conditions.

Interactions of EC and AC is important during development and maintenance of blood barrier [25]. To examine the effects of glucose conditions on these interactions, migration of EC was assessed using the transwell migration assay in the presence of conditioned medium collected from AC cultured under different glucose conditions. Conditioned medium from AC under high glucose conditions inhibited EC migration compared with normal glucose or osmolarity control conditions (Fig. 2D).

**Figure 3:**
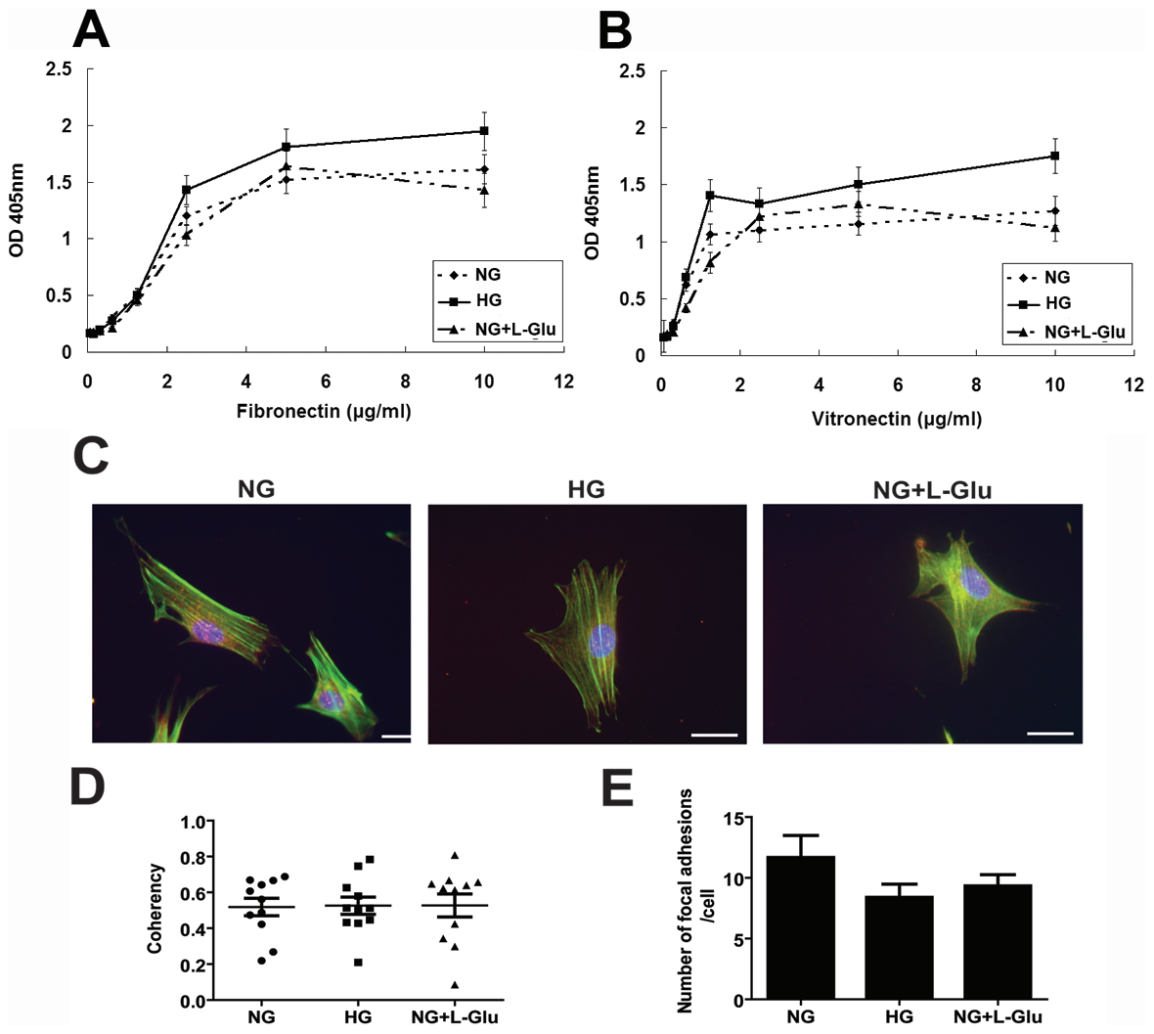
High glucose conditions enhanced the adhesion of retinal AC to extracellular matrix (ECM) proteins. Adhesion of retinal AC to fibronectin (A) and vitronectin (B) was determined by measuring the number of adherent cells in each well coated with different concentration of ECM proteins. Number of adherent cells was quantified by measuring the cellular phosphatase activity as described in Methods. Data are presented as mean ± SEM. n = 3; *p<0.05 (NG vs. HG). No significant adhesion to collagen I or laminin was observed (not shown). The impact of different glucose conditions on formation of actin stress fibers and focal adhesions in retinal AC. (C) Examination of actin stress fibers and focal adhesions in retinal AC. Retinal AC were stained with anti-vinculin (red), phalloidin (green), and DAPI (×630). Scale bar  = 20 µm. (D) Coherency of stress fibers was assessed using image J software. Data are presented as Mean ± SEM. (E) The number of focal adhesions was quantified per cell. Cells (5 to 10) in each condition were analyzed. Data are presented as Mean ± SEM. n ≥5; *p*>0.05 (NG vs. HG).

The migratory defects observed in retinal AC under high glucose suggested that alterations in cell adhesive mechanisms may exist. We next evaluated the adhesion of retinal AC cultured under different glucose conditions to various extracellular matrix proteins under normal or high glucose conditions. Figures 3A, B show that retinal AC adhered to fibronectin and vitronectin, and high glucose increased their adhesion to these ECM proteins. Very few cells adhered to collagen I or laminin regardless of glucose conditions (not shown). We also examined organization of stress fiber and focal adhesions (Fig. 3C). Coherency of stress fibers and number of focal adhesions were not affected by various glucose conditions (Figure 3D,E).

### The Effects of High Glucose on PDGF-Mediated AC Migration

**Figure 4:**
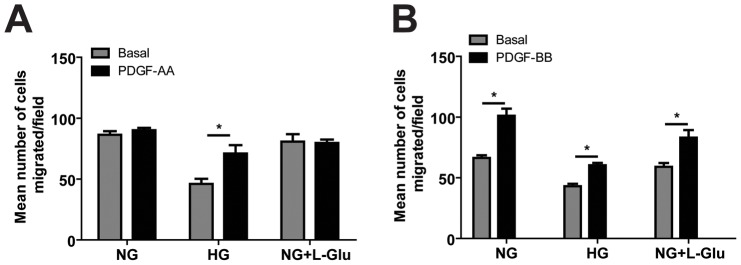
The effects of various glucose conditions on PDGF-mediated migration of retinal AC. Migration of retinal AC incubated under different glucose conditions for five days was determined using transwell assay. Transwell assay was performed with basal medium or medium containing PDGF-AA (A) or PDGF-BB (B) in lower compartment. Data are presented as mean ± SEM. n = 3; *p<0.05 (Basal vs. PDGF-AA or PDGF-BB). Please note PDFG-AA only enhanced the migration of AC under high glucose conditions. However, PDGF-BB enhanced AC migration under various glucose conditions.

The platelet derived growth factors (PDGF) play an important role in migration and proliferation of AC and inflammation [26]. To elucidate the effect of glucose conditions on PDGF-mediated AC migration, migratory activity of AC under various glucose conditions was investigated using a transwell migration assay with or without PDGF-AA or PDGF-BB. PDGF-AA had no effect on migration of AC cultured under normal glucose or osmolarity control conditions. However, PDGF-AA only enhanced the migration of AC cultured under high glucose conditions (Fig. 4A). The PDGF-BB enhanced migration of AC was not influenced under various glucose conditions (Fig. 4B).

### The Effects of High Glucose on Integrin Expression

**Figure 5:**
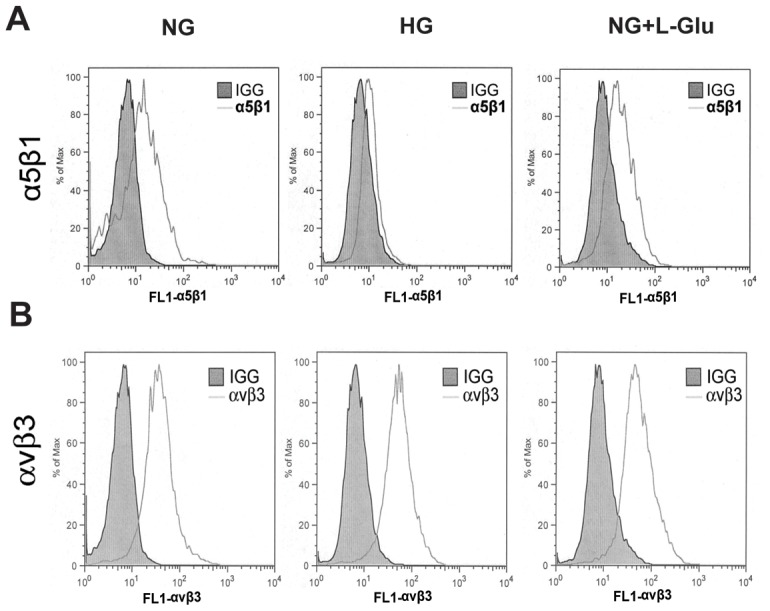
Expression of integrins in retinal AC under different glucose conditions. Integrins α5β1 (A) and αvβ3 (B) levels were determined by FACS analysis. Shaded areas show staining in the absence of primary antibody. Please note the dramatic decrease (2-fold) in mean florescence intensity of α5β1 integrin in AC under high glucose conditions compared to normal glucose and osmolarity control conditions (P<0.05, n = 3). This was mainly attributed with a nearly 2-fold decrease in the fluorescence intensity of α5 integrin, while the fluorescence intensity of β1 integrin did not change under various glucose conditions (not shown). The mean fluorescence intensity of αvβ3 was increased (1.5-fold) under high glucose or osmolarity control conditions compared to normal glucose conditions (P<0.05 n = 3), consistent with enhanced adhesion of AC to fibronectin and vitronectin under high glucose conditions.

An increase in adhesion of retinal AC to matrix proteins may be related to the enhanced expression and/or activity of integrins. We determined levels of α5β1 and αvβ3 integrins by FACS analysis. The α5β1integrin is a major receptor for fibronectin [27], while αvβ3 is a major receptor for vitronectin [28]. The α5β1 integrin mean fluorescence intensity was decreased by approximately 2-fold under high glucose conditions compared with normal glucose and osmolarity control (Fig. 5A). The average fluorescence intensities were NG: 18.8±1.2 vs. HG: 10.5±1.0 (P<0.05) or NG+L-Glu: 18.3±1.5 (P>0.05), n = 3. This was mainly attributed to decreased levels of α5 integrin, while the level of β1 did not change under various glucose conditions (not shown). However, the mean fluorescence intensity of αvβ3 level was increased by 1.5-fold under high glucose and osmolarity control conditions compared with normal glucose conditions (Fig. 5B). The average fluorescence intensities were NG: 35.9±3.0 vs. 51.4±3.8 or NG+L-Glu: 54.2±4.0 (P<0.05), n = 3. This is consistent with increased adhesion to vitronectin and perhaps fibronectin. We were unable to examine the levels of αv and β3 integrins individually due to lack of suitable antibodies for mouse integrins in these cells.

**Figure 6:**
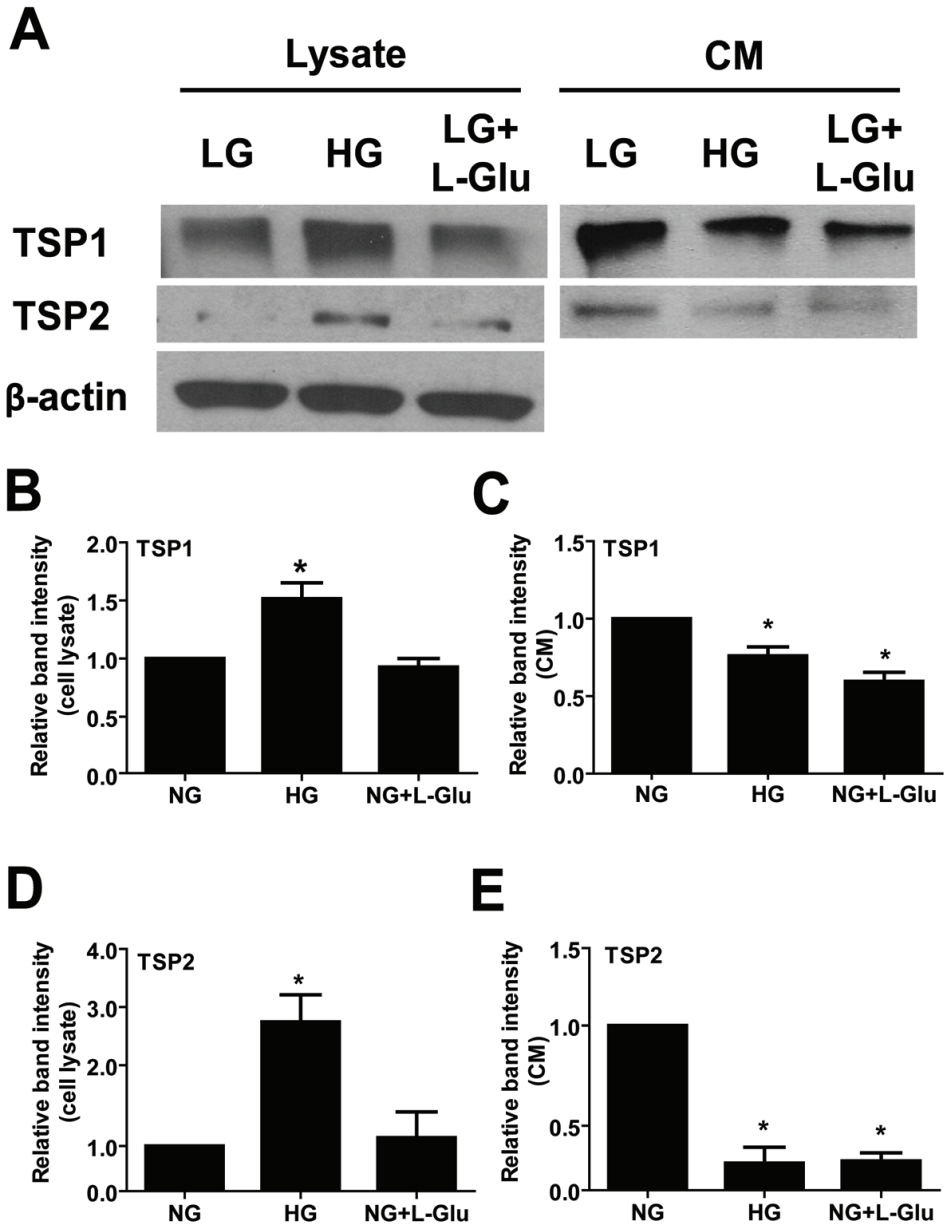
The effect of different glucose conditions on the expression and secretion of thrombospondins 1 and 2. (A) Levels of TSP1 and TSP2 in cell lysates and conditioned medium collected from retinal AC were determined by Western blot analysis. The β-actin was used as loading control for cell lysates. Quantification for band intensity is also shown. (B): TSP1 in cell lysates. (C): TSP1 in conditioned medium. (D): TSP2 in cell lysates. (E): TSP2 in conditioned medium. Data are presented as mean ± SEM. n = 3; *p<0.05 (NG vs. HG and NG vs. NG+L-Glu). Please note a significant decrease in the amount of TSP1 and TSP2 secreted by retinal AC under high glucose and osmolarity control conditions. No differences were observed in the levels of fibronectin, osteopontin, SPARC, periostin, and tenascin-C (not shown).

We also examined the impact of high glucose on production of various extracellular matrix (ECM) proteins. Thrombospondin-1 and -2 (TSP1 and TSP2) are extracellular matrix proteins and produced by various cell types including AC. TSP1 and TSP2 have anti-angiogenic activity, and their production by AC is essential for synaptogenesis of retinal ganglion cells [29]. These activities are mediated by various receptors [29], [30], [31]. Although, high glucose conditions increased cell-associated TSP1 and TSP2 (Fig. 6B,D), the secreted forms of TSP1 and TSP2 from retinal AC under high glucose and osmolarity control were decreased compared with normal glucose conditions (Fig. 6C,E). We also found minimal changes in the levels of other ECM proteins including fibronectin, osteopontin, SPARC, periostin, and tenascin C (not shown). Thus, alterations in TSP production of AC under osmotic stress may contribute to neuronal defects associated with diabetes.

### Alterations in Intracellular Signaling Pathways of Retinal AC under High Glucose Conditions

**Figure 7:**
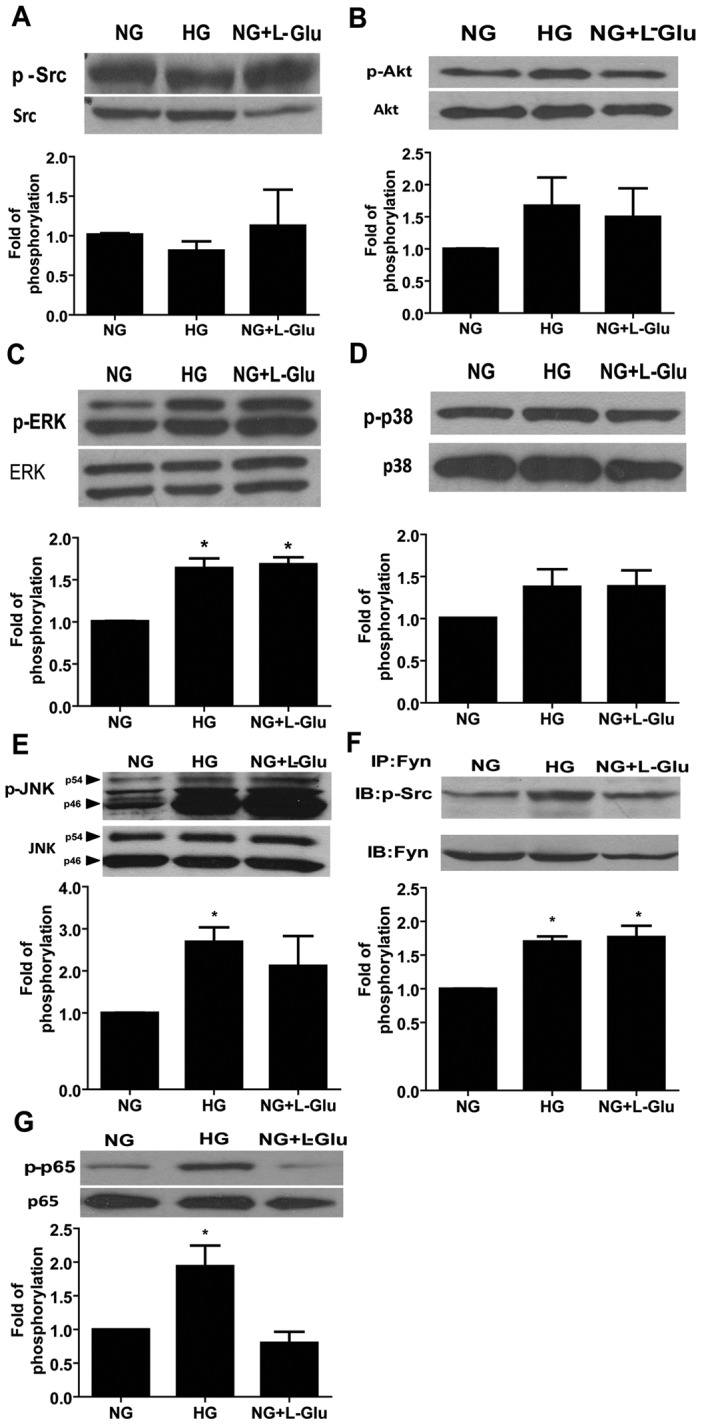
The impact of high glucose conditions on various down-stream signaling pathways. The status of various signaling pathways in retinal AC under various glucose conditions was determined as described in Methods. Levels of active Src (A), Akt (B), ERK (C), p38 (D), JNK1 (E), Fyn (F) and p65 (G) in retinal AC under different glucose conditions were determined by Western blot analysis from equal amounts of cell lysates. The total level for each protein is shown in the lower part of each panel. The quantitative assessment of the data for each blot is shown below the blot. Data are presented as mean ± SEM. n = 3; *p<0.05 (NG vs. HG and NG vs. NG+L-Glu). Levels of active Fyn (F) in retinal AC under different glucose conditions was determined by Western blot analysis of immunoprecipitates from equal amounts of retinal AC lysates.

The decreased AC migration observed under high glucose conditions may be related to alterations in intracellular signaling pathways. The Src kinase, Akt, and MAP kinase signaling pathways play important roles in cell survival, migration, and proliferation. We observed that Src phosphorylation was not affected in AC under high glucose conditions (Fig. 7A). In addition, activity of Akt was not affected by different glucose conditions (Fig. 7B). The phosphorylation of MAPK/ERKs was increased in retinal AC under high glucose and osmolarity control compared with normal glucose conditions (Fig. 7C). Phosphorylation of p38 MAPK was not affected by glucose conditions (Fig. 7D). Phosphorylation of JNK/MAP kinase was augmented under high glucose compared with normal glucose and osmolarity control conditions (Fig. 7E). In addition, Western blot analysis of Fyn immunoprecipitates from cell lysates using a specific active Src antibody showed increased activation of Fyn in retinal AC under high glucose and osmolarity control conditions compared to normal glucose conditions (Fig. 7F). Nuclear factor (NF)-κB has a crucial role in responding to cellular stress and production of inflammatory mediators. To determine the effect of high glucose conditions on the NF-κB signaling pathway, the level of phosphorylated p65 was determined by Western blot analysis. High glucose conditions increased phosphorylation of p65 compared with normal glucose and osmolarity control conditions (Fig. 7G). Thus, high glucose conditions resulted in sustained activation of MAPK/ERKs, JNK, and NF-κB in retinal AC.

### The Effects of High Glucose on Production of Inflammatory Cytokines

**Figure 8:**
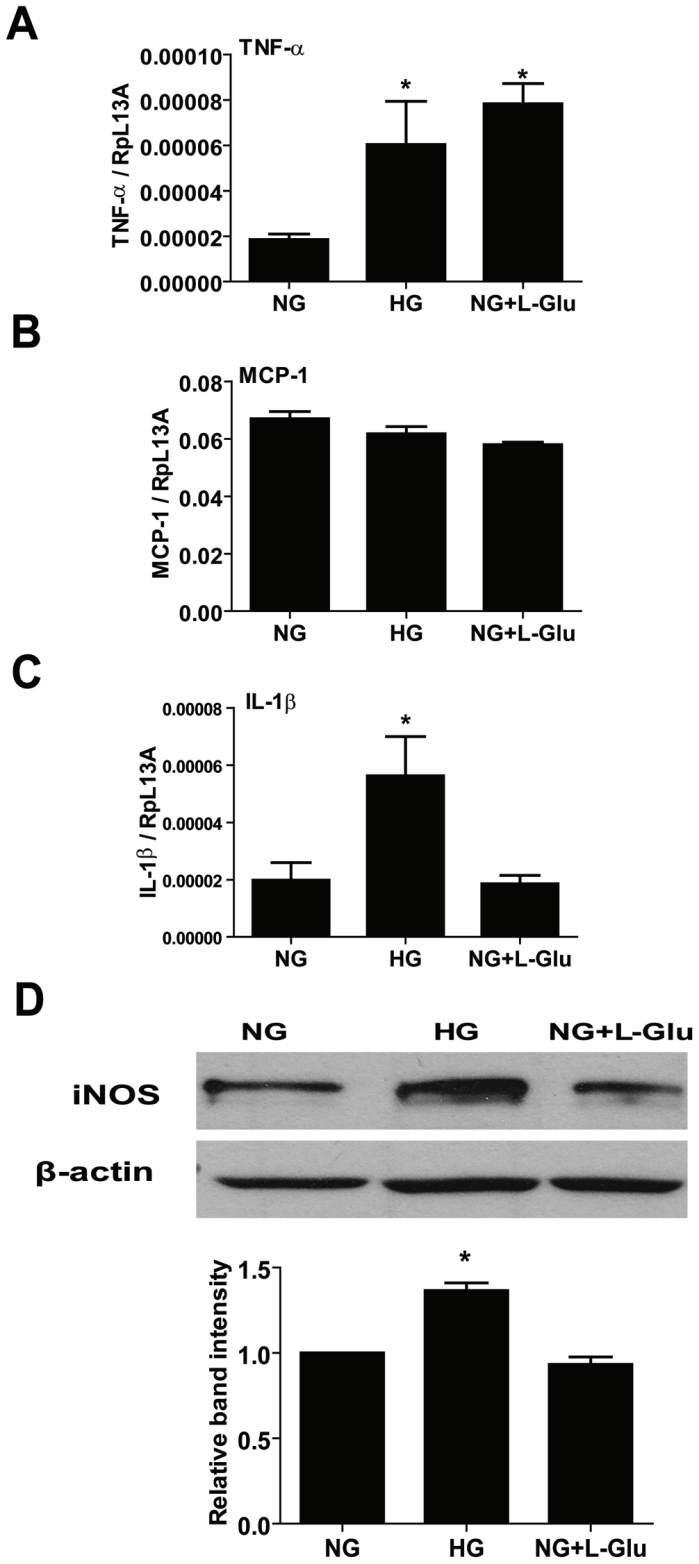
Increased expression of inflammatory cytokines in retinal AC under different glucose conditions. The mRNA levels for inflammatory cytokines in retinal AC under different glucose conditions were investigated by quantitative real time PCR. TNF-α (A), MCP-1 (B) and IL-1β (C) levels are shown. n = 5; *p<0.05 (NG vs. HG and NG vs. NG+L-Glu). (D) The iNOS protein level was determined by Western and the quantification of the data is also shown. The β-actin was used as loading control. Data are presented as mean ± SEM. n = 3; *p<0.05 (NG vs. HG and NG vs. NG+L-Glu).

Migration of AC is related with the level of inflammatory cytokines [32]. We next determined the mRNA levels of inflammatory cytokines including tumor necrosis factor-α (TNF-α), interleukin-1β (IL-1β) and monocyte chemotactic protein-1 (MCP-1) by quantitative real time PCR. High glucose and osmolarity control conditions increased the TNF-α level compared with normal glucose conditions (Fig. 8A). The MCP-1 level was not affected by glucose conditions (Fig. 8B). High glucose conditions increased the mRNA level of IL-1β compared with normal glucose and osmolarity control (Fig. 8C).

Inducible nitric oxide synthase (iNOS) is involved in inflammation and its expression is up-regulated by inflammatory mediators including TNF-α and IL-1β [33]. We next examined the iNOS level in retinal AC under various glucose conditions. High glucose conditions increased the iNOS level compared with normal glucose and osmolarity control conditions (Fig. 8D). Thus, high glucose conditions promote the inflammatory characteristics of AC.

### Increased Nuclear Localization of Nrf2 in AC under High Glucose Conditions

**Figure 9:**
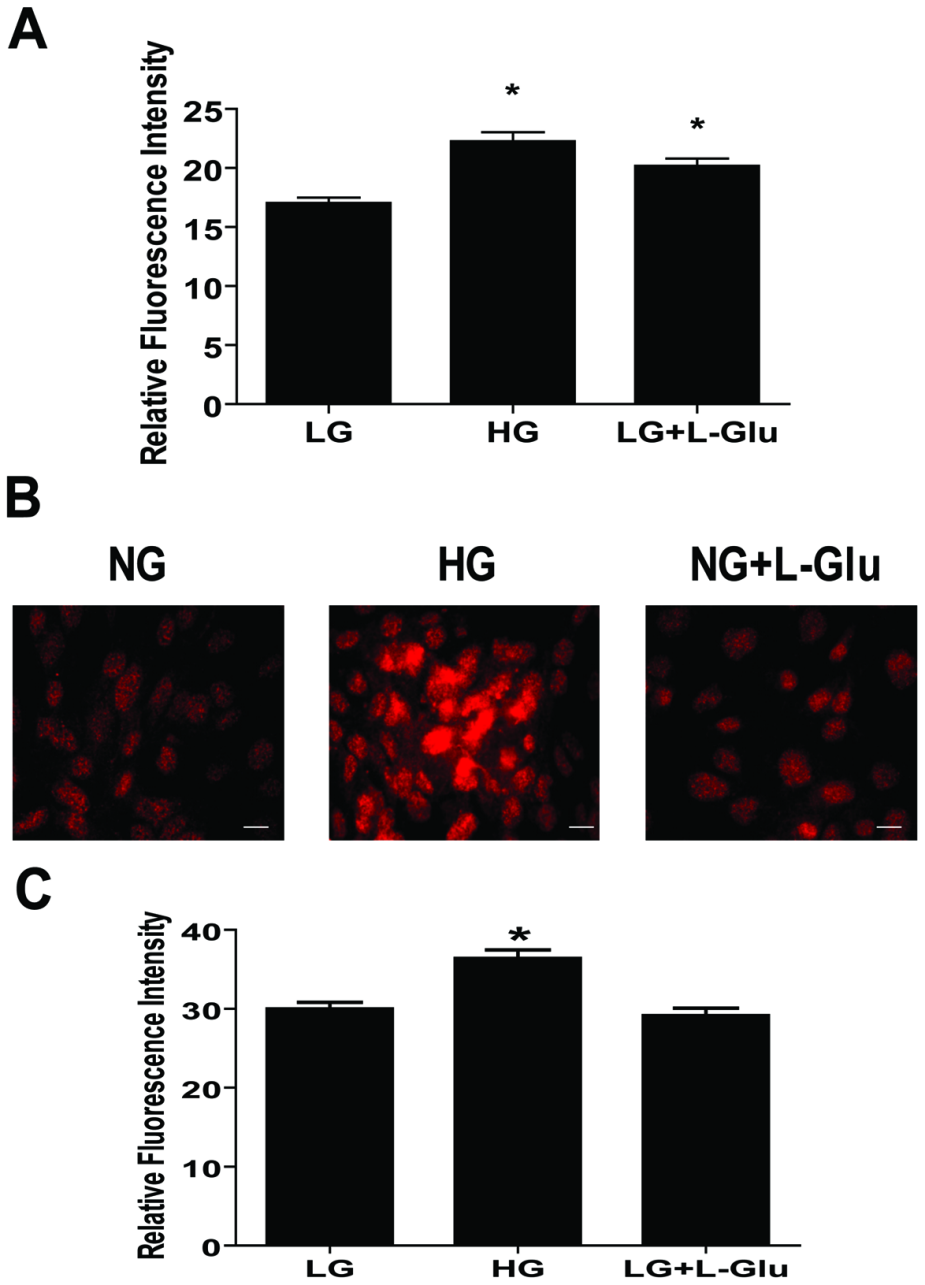
The impact of high glucose conditions on the level of ROS and nuclear localization of Nrf2 in retinal AC. (A) Retinal AC cultured under high glucose or osmolarity control exhibited a significantly elevated level of ROS compared to normal glucose conditions. Data are presented as mean ± SEM. n≥60; *p<0.05 (NG vs. HG and NG vs. NG+L-Glu). (B) Nuclear localization of Nrf2 in retinal AC under different glucose conditions was examined by immunofluorescence staining. Scale bar indicates 20 µm. (C) Nuclear localization of Nrf2 was assessed by measuring intensity of fluorescence in cell nucleus as described in Methods. Data are presented as mean ± SEM. n≥150 cells; *p<0.05 (NG vs. HG).

High glucose conditions induce reactive oxygen species (ROS) production in brain AC [34]. We next determined ROS levels by DHE staining of retinal AC cultured under various glucose conditions. High glucose and osmolarity control conditions increased ROS production in retinal AC (Fig. 9A). Nuclear factor erythroid 2-related factor 2 (Nrf2) is a transcription factor which regulates transcription of ROS-sensitive genes under oxidative stress. Nrf2 is translocated into the nucleus to induce transcription of genes, which are involved in the protective mechanisms against oxidative stress [16]. Nuclear localization of Nrf2 was examined by immunofluorescence staining. High glucose conditions increased nuclear localization of Nrf2 compared with normal glucose and osmolarity control conditions (Fig. 9B). Quantification of the signal intensity in cell nucleus is shown in Figure 9C. Western blot analysis demonstrated that the total level of Nrf2 was not affected under various glucose conditions (not shown).

**Figure 10:**
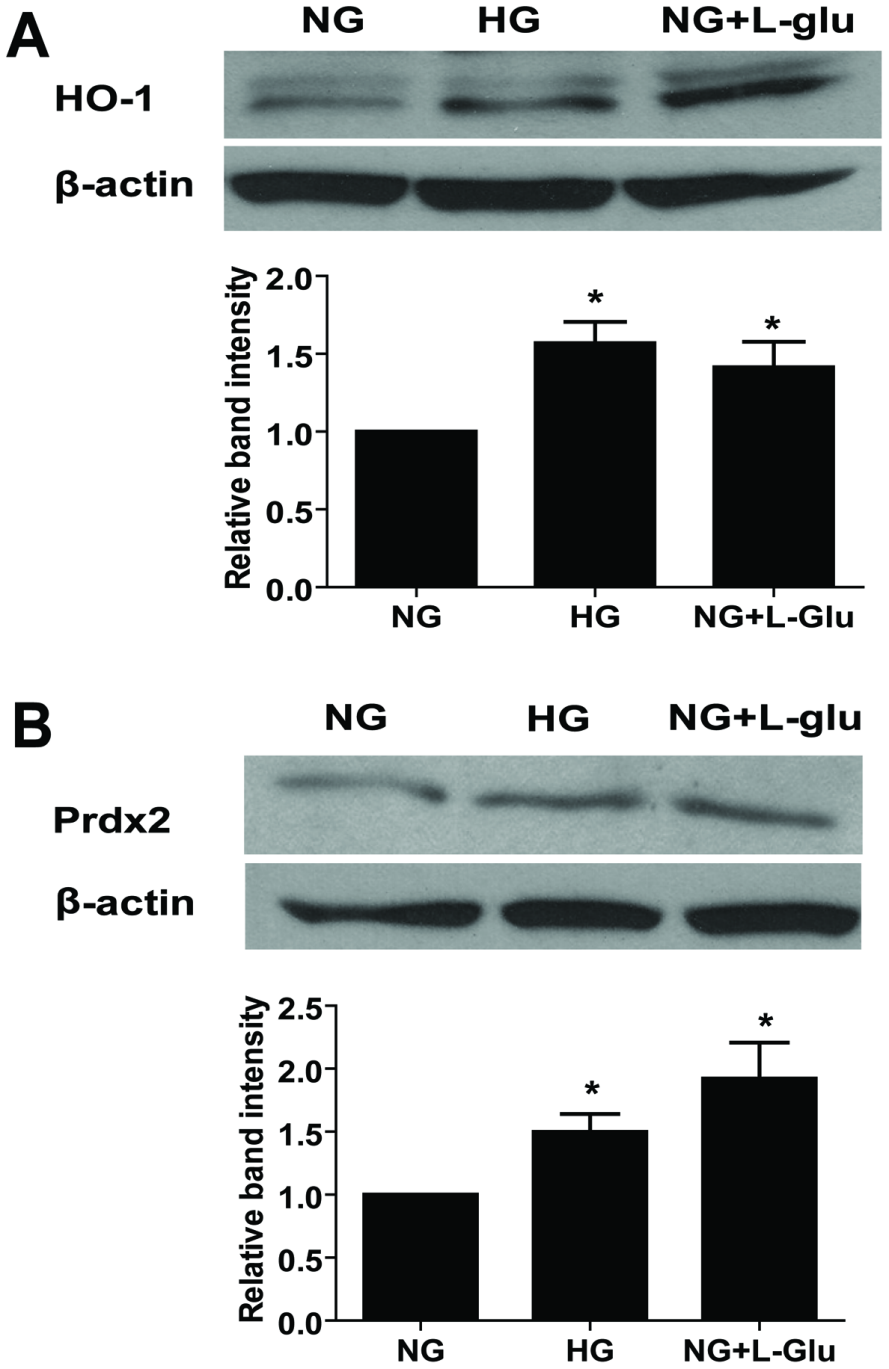
The effect of different glucose conditions on the expression of antioxidant enzymes. The expression of HO-1 (A) and Prdx2 (B) was determined by Western blot analysis. The quantification of data is also shown. The β-actin was used as loading control. Data are presented as mean ± SEM. n = 3; *p<0.05 (NG vs. HG and NG vs. NG+L-Glu).

We next asked whether increased Nrf2 activity, in conjunction with its increased nuclear localization, resulted in increased expression of its target genes including peroxiredoxin-2 (Prdx2) and heme oxygenease-1 (HO-1). Western blot analysis demonstrated increased expression of Prdx2 and HO-1 under high glucose and osmolarity control compared with normal glucose conditions (Fig. 10A, B).

### Effects of Antioxidant NAC on the Migration of Retinal AC and Capillary Morphogenesis of Retinal EC

**Figure 11:**
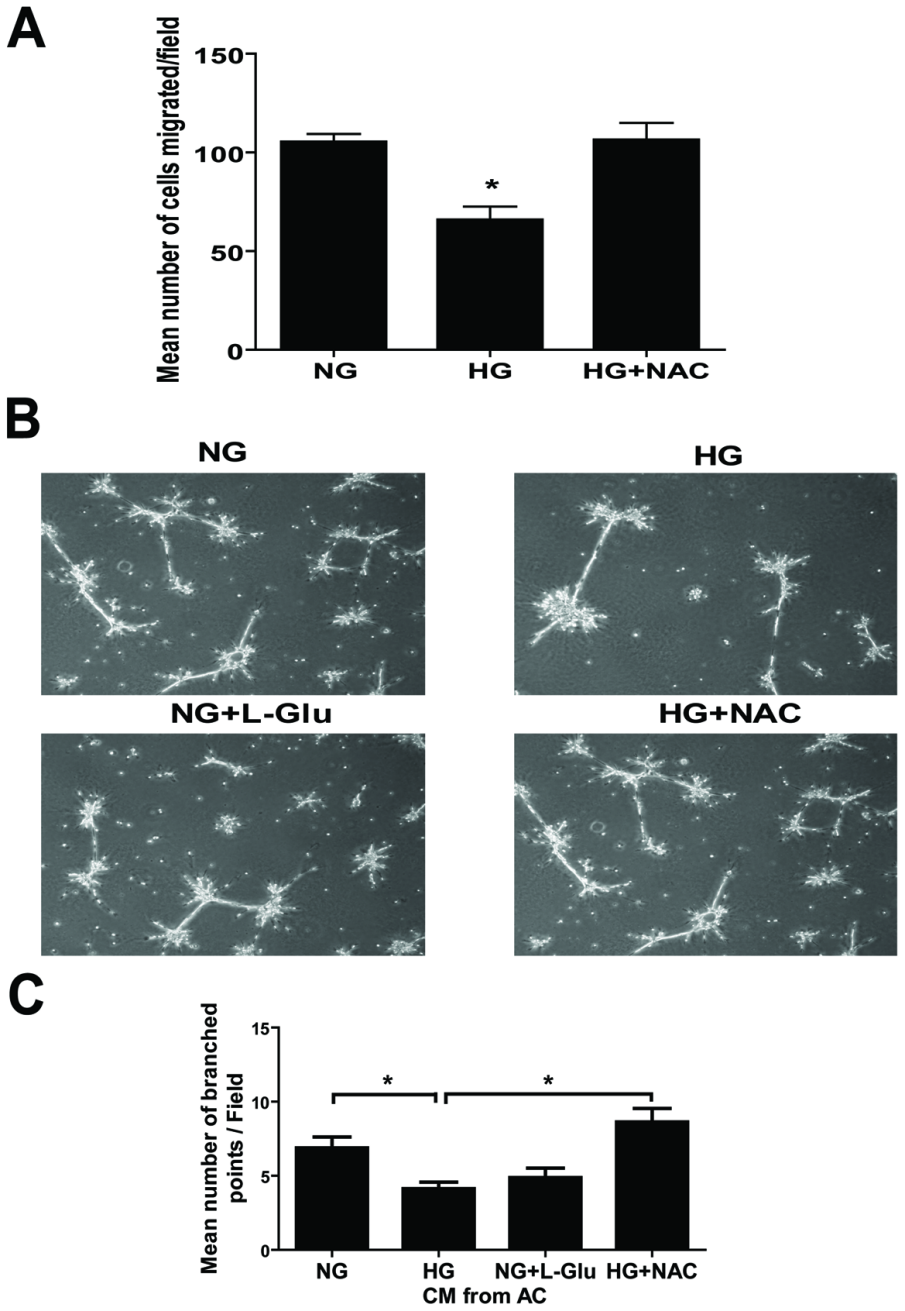
Effects of NAC on the migration of retinal AC under different glucose conditions and capillary morphogenesis of retinal EC. (A) Incubation of retinal AC cultured under high glucose with NAC restored basal migration. Data are presented as mean ± SEM. n = 3; *p<0.05 (NG vs. HG). (B) Retinal EC were resuspended in conditioned medium collected from retinal AC incubated under different glucose conditions with or without NAC and plated on Matrigel to analyze capillary morphogenesis. (C) The quantitative assessment of capillary morphogenesis of retinal EC. Data are the mean number of branch points from 8 high-power fields (×100) ± SEM. *p<0.05; n = 8 (NG vs. HG and HG vs. HG+NAC). NAC had no effect on capillary morphogenesis of retinal EC in the absence of AC conditioned medium (not shown).

We showed that high glucose conditions attenuated migration of retinal AC and increased oxidative stress of retinal AC. To determine whether oxidative stress induced by high glucose contributes to the attenuation of migration, retinal AC were incubated under high glucose conditions with or without the antioxidant NAC for five days. NAC restored the migration of retinal AC under high glucose conditions to that observed in AC cultured under normal glucose conditions (Fig. 11A).

We next asked whether the conditioned medium collected from retinal AC under various glucose conditions could affect capillary morphogenesis of retinal EC. Retinal EC were resuspended in conditioned medium prepared from AC and plated on Matrigel. Conditioned medium from AC under high glucose conditions inhibited capillary morphogenesis of EC compared with conditioned medium from AC cultured under normal glucose conditions (Fig. 11B). Capillary morphogenesis was rescued when conditioned medium was prepared from AC cultured under high glucose conditions with NAC. The quantitative assessment of the data is shown in Figure 11C. Thus, increased oxidative stress in AC contributes to the attenuation of their migration and capillary morphogenesis of EC under high glucose conditions.

## Discussion

Here we determined the impact of high glucose conditions on retinal AC properties. High glucose conditions did not affect retinal AC apoptosis, but increased the percentage of retinal AC undergoing active DNA synthesis. High glucose conditions also increased the expression of GFAP in retinal AC, and attenuated the migration and morphogenesis of AC on Matrigel. These changes were associated with the activation of signaling molecules involved in migration and proliferation, and increased mRNA level of inflammatory cytokines including IL-1β and TNF-α. In addition, high glucose conditions resulted in increased oxidative stress concomitant with the activation of Nrf2 and expression of antioxidant genes, Prdx2 and HO-1. The conditioned medium from AC under high glucose conditions also inhibited the migration and capillary morphogenesis of retinal EC. Capillary morphogenesis was restored when astrocytes were incubated with NAC under high glucose conditions. Together, our results suggest that high glucose conditions affect proliferation, adhesion, and migration of retinal AC through increased oxidative stress and production of inflammatory mediators.

The effect of diabetic conditions on the apoptosis of vascular cells including retinal AC has been subject of numerous studies. In the retina of diabetic rats, the rate of neuronal apoptosis was elevated compared with non-diabetic rats. However, the apoptosis rate of retinal AC in diabetic mice was similar to that of non-diabetic mice [35]. These results are consistent with our observation that high glucose conditions did not increase the rate of apoptosis in retinal AC. Thus, apoptosis of retinal AC is not affected under high glucose conditions, unlike retinal pericytes [36]. This may be attributed to specific activation of Nrf2 and enhanced expression of anti-oxidant enzymes in AC cultured under high glucose conditions, which does not occur in retinal pericytes [36].

High glucose conditions elevated ROS levels and oxidative stress in retinal AC, and resulted in the activation of cellular defense mechanisms. Nrf2 is a transcription factor that regulates the expression of antioxidant enzymes including Prdx2 and HO-1 in response to oxidative stress. Oxidative stress also induces phosphorylation of Fyn kinase, which can increase phosphorylation of Nrf2 leading to its degradation [16]. Here we showed that high glucose conditions increased p-Fyn levels in response to oxidative stress. However, we did not observe a significant difference in the total levels of Nrf2 in AC cultured under various glucose conditions (not shown).

To activate transcription of antioxidant enzyme, Nrf2 is translocated into the nucleus and binds to a cis-acting antioxidant responsive element (ARE) in antioxidant genes [16], [37]. High glucose conditions increased nuclear translocation of Nrf2 in retinal AC. These changes were concomitant with increased levels of antioxidant enzymes Prdx2 and HO-1. Heme oxygenase-1 is an enzyme that responds to stress and the rate-limiting enzyme in heme catabolism. Induction of HO-1 in diabetic retinopathy has protective roles by anti-inflammatory, anti-apoptosis and anti-proliferative effects [38]. Prdx2 is a cellular peroxidase that reduces H_2_O_2_ and prevents inactivation of redox-sensitive signaling pathways [39]. Prdx2 protects against apoptosis in retinal photoreceptor cells [40]. Thus, up-regulation of antioxidant enzyme including Prdx2 and HO-1 might protect retinal AC against oxidative stress induced under high glucose conditions. However, recently a role for Nrf2 in increased oxidative stress and proinflammatory responses has been demonstrated through stimulation of transcription factor Kruppel-like factor 9 (Klf9) [41] and activation of inflammasome and production of IL-1β [42]. Whether Klf9 is expressed in retinal AC, and if its expression is regulated under high glucose conditions contributing to the increased ROS production and activation of inflammasome remains to be determined.

GFAP has crucial roles in the intermediate filament network formation and is an essential component of the AC cytoskeleton [22]. Expression of GFAP is strongly enhanced in wound area of AC, and is an indicator of stress and damage in astrocytes [43]. High glucose conditions increased the GFAP level in retinal AC, thus, implying that retinal AC are stressed/damaged under high glucose conditions. Furthermore, GFAP is also involved in retinal AC migration [22]. Migration of retinal AC is essential for tissue repair in response to pathogenic events [27], and is inhibited by increased expression of GFAP [44]. The organization of actin cytoskeleton and formation of actin stress fibers and focal adhesions can also modulate cell migration. Different glucose conditions did not affect the formation of actin stress fibers and focal adhesions in retinal AC. Thus, the attenuation of retinal AC migration might result from increased GFAP levels and perhaps stiffness of AC under high glucose conditions. These observations are also consistent with enhanced adhesion of retinal AC to ECM proteins including fibronectin and vitronectin under high glucose conditions, and may be further influenced by production of inflammatory mediators and oxidative stress.

The antioxidant NAC restored the normal migration of retinal AC and capillary morphogenesis of retinal EC incubated with AC conditioned medium prepared under high glucose conditions, thus, supporting an important role for oxidative stress in AC adhesive and migratory properties under high glucose conditions. We recently showed that enhanced production of inflammatory mediators is a primary response in retinal pericytes exposed to high glucose, and leads to increased oxidative stress in these cells [36]. Inflammatory cytokines including IL-1β and TNF-α promote adhesion of AC to fibronectin. IL-1β inhibits migration, while TNF-α promotes migration of AC. However, combination of IL-1β and TNF-α inhibits migration of AC [32]. Here we observed that high glucose conditions increased mRNA levels for IL-1β and TNF-α. Thus, attenuated migration of retinal AC under high glucose condition could be attributed, at least in part, to the enhanced IL-1β expression and increased adhesion under high glucose conditions.

Conditioned medium prepared from retinal AC cultured under high glucose conditions also inhibited the migration and capillary morphogenesis of retinal EC. We recently demonstrated that IL-1β and TNF-α inhibit the migration and capillary morphogenesis of retinal EC under normal glucose conditions [45]. Thus, attenuation of migration and capillary morphogenesis of retinal EC by conditioned medium collected from retinal AC under high glucose conditions may also be attributed to increased level of IL-1β and/or TNF-α in retinal AC, and is consistent with the activation of NF-κB in AC under high glucose conditions [46].

In the central nervous system AC are strategically located and control both vascular and neuronal functions. Thus, their alterations under various pathological conditions may impact both neuronal and vascular function. An important neuronal function of AC is mediated though their ability to promote and maintain the synaptic function of neurons, at least in part through production of TSP1 and TSP2 [29]. Incubation of AC with high glucose did not significantly affect the overall levels of TSP1 and TSP2 produced by these cells. However, we observed a significant increase in the level of cell associated TSP1 and TSP2 under high glucose conditions, while the levels of secreted TSP1 and TSP2 were diminished in the conditioned medium. Thus, high glucose levels may interfere with posttranslational processing of TSP1 and TSP2, and as a result their potential signaling mechanisms interfering with neuronal function as occurs in diabetes [47].

Astrocytes activate repair processes by undergoing proliferation when they are damaged by environmental stimuli [43]. Proliferation of AC is mediated by the activation of ERK, and Fas/CD95 may be involved in the activation of ERK [48]. High glucose conditions increased the percentage of retinal AC undergoing active DNA synthesis. Moreover, phosphorylation of ERK was increased under high glucose conditions. These results suggested that enhanced proliferation of retinal AC under high glucose conditions is mediated by the activation of ERK. Fas/CD95 interactions activate ERKs in AC [48]. However, how high glucose conditions affect Fas/CD95 expression, ERK activation, and enhanced AC proliferation are subject of future investigation in our laboratory.

In summary, our studies demonstrate that exposure of retinal AC to high glucose conditions results in attenuation of migration, increased number of cells undergoing active DNA synthesis, and adhesion. We showed that high glucose conditions increased expression of inflammatory cytokines including IL-1β and TNF-α, and oxidative stress in retinal AC. Conditioned medium collected from retinal AC under high glucose conditions impacted migration and capillary morphogenesis of retinal EC. Retinal AC responded to oxidative stress by elevating the levels of antioxidant enzymes including HO-1 and Prdx2 through nuclear translocation of transcription factor Nrf2. Collectively our data suggest that high glucose conditions result in dysfunction of retinal AC through increased production of inflammatory mediators and oxidative stress, thus impacting their proliferation, adhesion, and migration.
